# Validation of Suitable Reference Genes for Quantitative Gene Expression Analysis in *Panax ginseng*

**DOI:** 10.3389/fpls.2015.01259

**Published:** 2016-01-12

**Authors:** Meizhen Wang, Shanfa Lu

**Affiliations:** Medicinal Plant Cultivation Research Center, Institute of Medicinal Plant DevelopmentBeijing, China

**Keywords:** *Panax ginseng*, RT-qPCR, reference gene, mlncRNA, normalization, validation, heat stress

## Abstract

Reverse transcription-qPCR (RT-qPCR) has become a popular method for gene expression studies. Its results require data normalization by housekeeping genes. No single gene is proved to be stably expressed under all experimental conditions. Therefore, systematic evaluation of reference genes is necessary. With the aim to identify optimum reference genes for RT-qPCR analysis of gene expression in different tissues of *Panax ginseng* and the seedlings grown under heat stress, we investigated the expression stability of eight candidate reference genes, including elongation factor 1-beta (*EF1-*β), elongation factor 1-gamma (*EF1-*γ), eukaryotic translation initiation factor 3G1 (*IF3G1*), eukaryotic translation initiation factor 3B (*IF3B*), actin (*ACT*), actin11 (*ACT11*), glyceraldehyde-3-phosphate dehydrogenase (*GAPDH*), and cyclophilin ABH-like protein (*CYC*), using four widely used computational programs: geNorm, Normfinder, BestKeeper, and the comparative ΔCt method. The results were then integrated using the web-based tool RefFinder. As a result, *EF1-*γ, *IF3G1*, and *EF1-*β were the three most stable genes in different tissues of *P. ginseng*, while *IF3G1, ACT11*, and *GAPDH* were the top three-ranked genes in seedlings treated with heat. Using three better reference genes alone or in combination as internal control, we examined the expression profiles of *MAR*, a multiple function-associated mRNA-like non-coding RNA (mlncRNA) in *P. ginseng*. Taken together, we recommended *EF1-*γ/*IF3G1* and *IF3G1*/*ACT11* as the suitable pair of reference genes for RT-qPCR analysis of gene expression in different tissues of *P. ginseng* and the seedlings grown under heat stress, respectively. The results serve as a foundation for future studies on *P. ginseng* functional genomics.

## Introduction

Real-time quantitative reverse-transcriptase polymerase chain reaction (RT-qPCR) has been widely used as a powerful technique to determine gene expression levels, due to its high sensitivity, specificity and reproducibility (Bustin, 2002; Derveaux et al., 2010). It must be noted that the accuracy of RT-qPCR results strongly depends on the stability of reference gene(s) used for data normalization. In plants, several reference genes, such as those encoding actins, ubiquitins, and rRNAs, are commonly used to standardize the semi-quantitative RT-PCR or RT-qPCR data (Guénin et al., 2009). In many cases, unfortunately, these reference genes were merely putatively housekeeping. They may be stable under some experimental conditions, but show a significant variance in other cases (Guénin et al., 2009). For example, *18S rRNA* showed the most stable expression in rice under various abiotic stresses, but not in different rice tissues tested (Jain et al., 2006). Therefore, it is requisite to determine suitable reference genes under specific experimental conditions before gene expression studies (Gutierrez et al., 2008; Guénin et al., 2009).

*Panax ginseng* C.A. Meyer is a perennial medicinal plant native to Asian. It is a member of the Araliaceae family and has been served as one of the most well-known herbs worldwide for human health improvement (Shibata, 2001). Triterpene saponins, named ginsenosides, are the main bioactive components in *P. ginseng*. It accounts for >4% of ginseng root dry weight (Shibata, 2001). Cultivation of ginseng is difficult due to long growth period (4–6 years), diseases, and special growth condition requirements, which makes *P. ginseng* mainly distributed in the northeast region of China. Large-scale cultivation of ginseng in Beijing is not successful yet. Ginseng growth has a strong correlation with the temperature. The growth rate of ginseng reaches a peak at 18°C and then declines with the temperature increases (Bi, 1985). Additionally, the highest ginseng yield occurs when the average temperature is 21°C in July, the hottest month in Liaoning province, China (Bi, 1985). Therefore, elucidation of the molecular mechanism of *P. ginseng* response to high temperature is of great importance and will foster the molecular breeding of heat resistant ginseng.

Next generation sequencing (NGS) technology has triggered *P. ginseng* molecular research, although it is still lagged far behind that of other crops, such as rice. Because of its complexity, the whole genome of *P. ginseng* has not been decoded. The chloroplast genomes of one Korean (*Panax schinseng* Nees) and four Chinese (Damaya, Ermaya, Gaolishen, and Yeshanshen) ginseng cultivars have been sequenced and comparatively analyzed (Kim and Lee, 2004; Zhao et al., 2015). To date, 17,773 *P. ginseng* expressed sequence tags (ESTs) have been deposited in the EST database at the National Center for Biotechnology Information (NCBI; February13, 2015). Several ginsenoside biosynthesis-related genes have been characterized. It includes squalene synthase gene *PgSS1* (Lee et al., 2004), squalene epoxidase gene *PgSQE1* (Han et al., 2010), dammarenediol synthase gene *DDS* (Han et al., 2006), dammarenediol 12-hydroxylase gene *CYP716A47* (Han et al., 2011), and protopanaxadiol 6-hydroxylase gene *CYP716A53v2* (Han et al., 2012). Many other candidate genes involved in ginsenoside biosynthesis were also identified by RNA-seq analysis of adventitious roots in two *P. ginseng* cultivars, Chunpoong and Cheongsun (Jayakodi et al., 2014). Additionally, a systematic identification of mRNA-like non-coding RNAs (mlncRNAs) in *P. ginseng* was performed, and a mlncRNA *MAR* probably involved in multiple metabolic pathways was characterized (Wang et al., 2015). These results provide the basis for a deeper insight into *P. ginseng* metabolic pathways. However, studies on the mechanism of *P. ginseng* response to heat stress have not been reported, with the exception of several heat-responsive miRNAs were identified (Wu et al., 2012).

*MAR* is accumulated to a much higher level in 4-year-old *P. ginseng* roots than that in 1-year-old roots. Hence, it is assumed to be associated with ginsenoside biosynthesis (Luo et al., 2003). Our recent study indicates that *MAR* is a long non-coding RNA, and it might be involved in multiple metabolic pathways through siRNA-dependent mechanisms (Wang et al., 2015). Tissue specific expression of *MAR* had been analyzed using the traditional reference gene *GAPDH* as internal control. It shows the highest expression in flower, followed by leaves, stems, roots, calli, and the least in adventitious roots. It is also induced by methyl jasmonate (MeJA; Wang et al., 2015).

Here, we evaluated the stability of eight candidate reference genes to identify the most suitable reference genes for RT-qPCR data normalization in two sets of *P. ginseng* samples, including different tissues and seedlings subjected to heat stress. Further, the expression patterns of our previously characterized *MAR* gene (Wang et al., 2015) in these samples were compared using three better reference genes alone or in combination as internal control. The optimum pairs of internal control genes were identified. It provides important basis for further studies on gene functions of *P. ginseng* in response to heat stress.

## Materials and methods

### Plant materials

*P. ginseng* C.A. Meyer cv. Shizhu, grown in a field nursery at Kuandian, Liaoning province, China, was used in this study.

The expression stability of putative reference genes was analyzed in two conditions: (1) among samples from different tissues (DT); (2) among samples from seedlings with or without heat treatment (SH). Group DT contains leaves, stems, flowers, roots collected from 5-year-old ginseng plants and embryogenic calli. Embryogenic calli were induced as described (Wu et al., 2012). Firstly, *P. ginseng* seeds were stratified in humidified sand for about 6 months at 10°C. After removing the pericarp, seeds were immersed in 70% ethanol for 30 s, and then transferred to 2% sodium hypochlorite for 30 min. Decontaminated seeds were rinsed three times in sterile distilled water. After that, cotyledon explants were dissected out and cultured on MS media supplemented with 1.0 mg/L 2,4-D for one month in the dark at 25–26°C. Embryogenic calli were then subcultured by regular 3-week intervals and cultured at 25–26°C under a 16/8-h (light/dark) photoperiod (24 mmol.m^−2^.s^−1^). All the tissues were frozen immediately in liquid nitrogen for total RNA extraction after collection.

Group SH contains ginseng seedlings with or without heat stress treatment. Seeds were sterilized as mentioned above and sowed in plastic pots containing nutrient soil and vermiculite mixed at 1:1 (v/v). After germination, the seedlings were grown in a climate controlled chamber with temperature at 25–26°C, humidity at 60–70%, and 16-h light/8-h darkness. For heat treatment, 1-month-old seedlings grown in 12 pots, with 30 ones per pot, were used. One half was grown under the same conditions with the exception of at 25–26°C for 6 (S6) or 12 h (S12). The other half was incubated at 37°C, at which the ginseng growth is inhibited but not lethal, for 6 (SH6) or 12 h (SH12). After treatment, the seedlings were collected and immediately stored in liquid nitrogen until use.

No specific permissions were required for the location of the field nursery. No endangered or protected species were involved in described field studies.

### Total RNA isolation, quality control, and first cDNA synthesis

Total RNA was extracted using Trizol reagent (Invitrogen, Carlsbad, USA) following the manufacturer's protocol, and then treated with RNase-free DNase I (TaKaRa, Shiga, Japan) for genomic DNA contamination removal.

RNA samples isolated were analyzed by 0.8% agarose gel electrophoresis, showing only the two rRNA subunits (28S and 18S) with no indication of RNA degradation (Figure S1A). Then, they were evaluated with a NanoDrop-2000C spectrophotometer (Thermo Scientific, Wilmington, DE) for concentration, quantity, and purity. All samples showed an absorbance ratio at 260/280 nm ranged from 2.02 to 2.10, reflecting pure and protein-free isolated RNA (Figure S1B). The four RNA samples from control and heat stressed seedlings (S6, S12, SH6, and SH12) were further examined using the Agilent 2100 Bioanalyzer (Agilent Technologies, Palo Alto, CA) with RIN values of above 8.0 (Figure S1C).

About 5 μg high-quality total RNA was reverse-transcribed into single-strand cDNA in a 20 μL reaction mix with SuperScript III Reverse Transcriptase (Invitrogen, Carlsbad, USA) and 50 ng random hexamers, and immediately stored at −20°C until use.

### Candidate reference gene selection, primer design, and RT-qPCR

To identify potential ginseng homologs of Arabidopsis genes commonly used as internal controls for gene expression, we searched ginseng assembled transcriptome database using Blastx with an *E* = 10^−5^. Eight candidates with near or longer than 500 bp in length were selected for further analysis. Sequence alignment was performed by T-coffee (Notredame et al., 2000). Specific primers pairs were designed for each candidate reference gene with Primer 3.0 software (version 0.4.0; http://bioinfo.ut.ee/primer3-0.4.0/). Primer pairs were designed as follows: primer length of 25 bp, product length of 150–250 bp, optimal *Tm* at 60°C, GC% between 40 and 60%. For the PCR efficiency analysis, equal amount of each cDNA sample was pooled. Then, standard curves were determined by using five-fold dilution series of pooled cDNA as a template using the linear regression model (Pfaffl et al., 2004). The efficiency was calculated by CFX Manager™ software in a Bio-Rad CFX96 Real-Time PCR System C1000 Thermal Cycler (Bio-Rad, Hercules, CA).

RT-qPCR amplification was carried out on the Bio-Rad CFX96 Real-Time PCR System C1000 Thermal Cycler (Bio-Rad, Hercules, CA) in triplicates. Reaction mixture (20 μL) contains 100 ng cDNA as a template, 10 μL 2X SYBR Premix Ex Taq II (TaKaRa, Shiga, Japan), 0.5 μM each specific forward and reverse primer (Table 1). The reaction condition was as follows: 95°C for 30 s, 35 cycles of 95°C for 10 s, 60°C for 10 s, and 72°C for 10 s.

**Table 1:** **Description of candidate reference genes and ***MAR*** in ***P. ginseng*****.

**Gene symbol**	**Gene description**	**GenBank accession number**	**Arabidopsis ortholog**	**Arabidopsis BlastX *E*-value**	**Primer sequences (5′–3′) (forward/reverse)**	**Primer *Tm* (°C)^a^**	**Product (bp)**	**Product *Tm* (°C)^b^**	***E*(%)^c^**	***R*^2c^**
**REFERENCE GENES**										
*ACT11*	Actin 11	KU215665	NP_187818.1	0	CCCGAGAGAAAGTATAGTGTATGGA	59.91	171	81.5	92.6	0.998
					TAGAGCTCTTCAACAACCACTTTTT					
*ACT*	Actin	KU215664	AAO42312.1	1e-55	AAAGATTTGGCATCACACCTTCTAC	60.95	203	83	111.4	0.990
					TACCTGTTGTACGACCACTAGCATA					
*EF1-β*	Elongation factor 1-beta	KU215660	NP_179402.1	1e-44	AAGAAGAAAGAGAGTGGGAAATCAT	60.02	158	82	112.5	0.997
					TTTATCCCATAACCAACAGGAAGTA					
*EF1-γ*	Elongation factor 1-gamma	KU215661	NP_176084.1	1e-60	ATCGCATTAAAGAGAGCACTAGGA	60.11	186	80.5	105.5	0.997
					CATGGTCCAAAAATATCTCTCTACG					
*IF3G1*	Eukaryotic translation initiation factor 3G1	KU215663	AAG53636.1	3e-34	GCGAACTAGAATATTTTCATCTCCA	59.90	208	79	113.9	0.994
					AGAAGAGAAGGGATAACAATTCCAT					
*IF3B*	Eukaryotic translation initiation factor 3B	KU215662	NP_568498.1	9e-109	CCTTGGTTACTGCTTCATTGAGTAT	59.94	198	81	110.2	0.997
					TGTTGTAAATTTTCCCCTGATGTAT					
*GAPDH*	Glyceraldehyde 3-phosphate dehydrogenase	AY345228	NP_172801.1	0	AAGGTTAAGGACAATAACACCCTTC	60.06	195	76.5	88.9	0.994
					AGGAGCTGAAATAACAACCTTCTTT					
*CYC*	Cyclophilin ABH-like protein	EF587913	NP_179251.1	2e-100	ATCTGAGTCTGGGTTGTGATAAAAG	60.00	175	85.5	117.3	0.994
					AACAACCAACCAAACCAAATTATTA					
**GENE**										
*MAR*	Multiple-function-associated mlncRNA	KU285606	N	N	GATGGTCCAGAGGATAGCATTAGTA	59.98	223	77.5	100.7	0.997
					TTATACATCACCTATCTTGGGTCGT					

a The primer melting temperature was calculated by Primer3 (v. 0.4.0).

b The product melting temperature was calculated by CFX Manager™ software.

c The RT-qPCR efficiency (E) and correlation coefficients (R^2^) were determined by CFX Manager™ software.

N, Not examined.

### Analysis of stability of candidate reference genes

The Ct-values generated by the CFX Manager™ software were used to analyze the gene expression levels. Four algorithms were used to evaluate the stability of the eight candidate reference genes: geNorm (Vandesompele et al., 2002), NormFinder (Andersen et al., 2004), BestKeeper (Pfaffl et al., 2004), and the comparative ΔCt method (Silver et al., 2006). RefFinder (http://www.leonxie.com/referencegene.php?type=reference) was used to compare and integrate the ranking the tested candidate reference genes.

### Validation of reference genes

To validate the reliability of the reference genes, the expression levels of a mlncRNA gene *MAR* (Wang et al., 2015) was analyzed using the top three ranking reference genes, as recommended by RefFinder, alone or with a combination for data normalization. By comparison, the least stable reference genes were also used. The relative expression of *MAR* was calculated using the 2^−ΔΔCq^ method (Livak and Schmittgen, 2001).

## Results

### Selection of candidate reference genes in *P. ginseng*

A total of nine RNA samples, which were divided into two groups (DT and SH), were used to evaluate the stability of reference gene expression. Group DT contains four samples extracted from roots, stems, leaves and flowers of 5-year-old *P. ginseng* plants, and one from embryogenic calli of *P. ginseng*. Group SH includes four samples from one-month-old *P. ginseng* seedlings treated with (SH6 and SH12) or without (S6 and S12) high temperature stress for 6 and 12 h. Based on Blastx alignment of *P. ginseng* ESTs (Wu et al., 2012) against *Arabidopsis* genes, eight candidate reference genes of *P. ginseng* were evaluated (Table 1). It includes genes encoding elongation factor 1-beta (*EF1-*β), elongation factor 1-gamma (*EF1-*γ), eukaryotic translation initiation factor 3G1 (*IF3G1*), eukaryotic translation initiation factor 3B (*IF3B*) (Figure S2), actin (*ACT*), actin11 (*ACT11*) (Figure S3), glyceraldehyde-3-phosphate dehydrogenase (*GAPDH*), and cyclophilin ABH-like protein (*CYC*). Among them, *ACT* and *ACT11* share 46.74% nucleotide sequence identity (Figure S3). RT-qPCR primers were designed in low sequence identity regions. The sizes of all PCR products were from 158 to 223 bp (Table 1).

### Amplification efficiency and specificity

**Figure 1:**
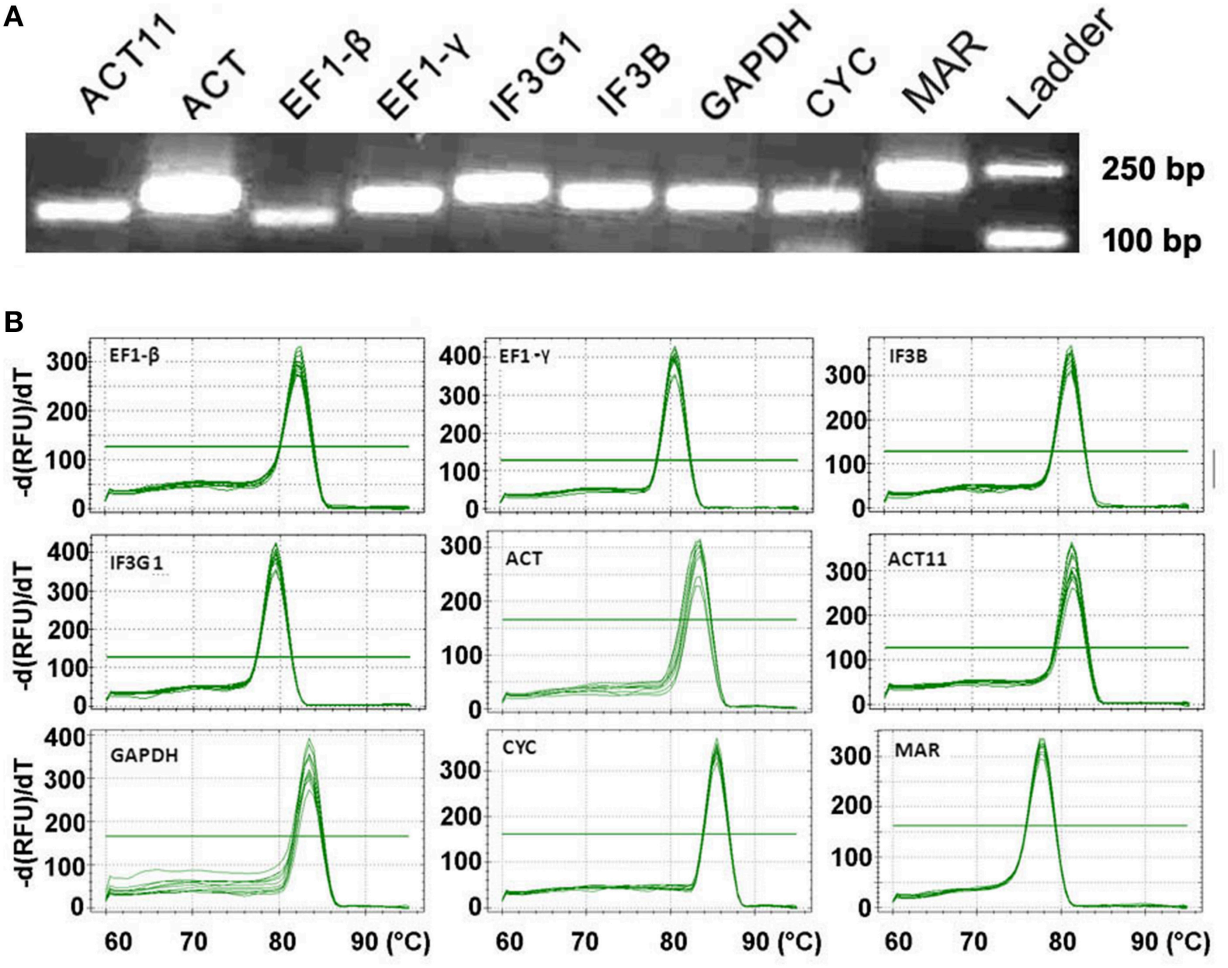
**RT-qPCR amplification specificity. (A)** Amplified fragments of eight candidate genes and target gene *MAR*. Agarose gel (3%) electrophoresis shows the specific PCR product with expected size for each gene. Equivalent cDNA of all nine samples was pooled and used as a template. The 250 and 100 bp DNA ladders were shown. **(B)** Melting curves for eight candidate reference genes and *MAR* with single peak. Equivalent cDNA of all nine samples was pooled and used as a template. Temperature is displayed in the x axis, and the derivative reporter signal is displayed in the y axis.

High RT-qPCR amplification efficiency is usually correlated with the robust and precise results of gene expression assays (Bustin et al., 2009). The *Tm* for all PCR products ranged from 76.5°C (*GAPDH*) to 85.5°C (*CYC*), which was in the range expected based on their length and the CG composition. The efficiency (E) for the eight candidate reference genes varied from 88.9% (*GAPDH*) to 117.3% (*CYC*) (Table 1). The amplification specificity of each candidate reference gene was verified by agarose gel electrophoresis of the products of PCR using pooled cDNA as a template. The results showed only one single band with expected size (Figure 1A). Gene-specific amplification of each gene was also confirmed by single peak in the melting curve generated during RT-qPCR (Figure 1B). No signal was detected in the reverse transcription negative control and the non-template control. Considering the high sequence similarity of *ACT* and *ACT11* (Figure S3), the PCR products using each primer pair were cloned and sequenced, respectively. As a result, all the 48 randomly picked amplicons of each gene are specific even though there are only four mismatches in the primers, confirming the high specificity. These results suggest that the designed primers are suitable for RT-qPCR analysis of gene expression.

### Expression profiling of candidate reference genes

**Figure 2:**
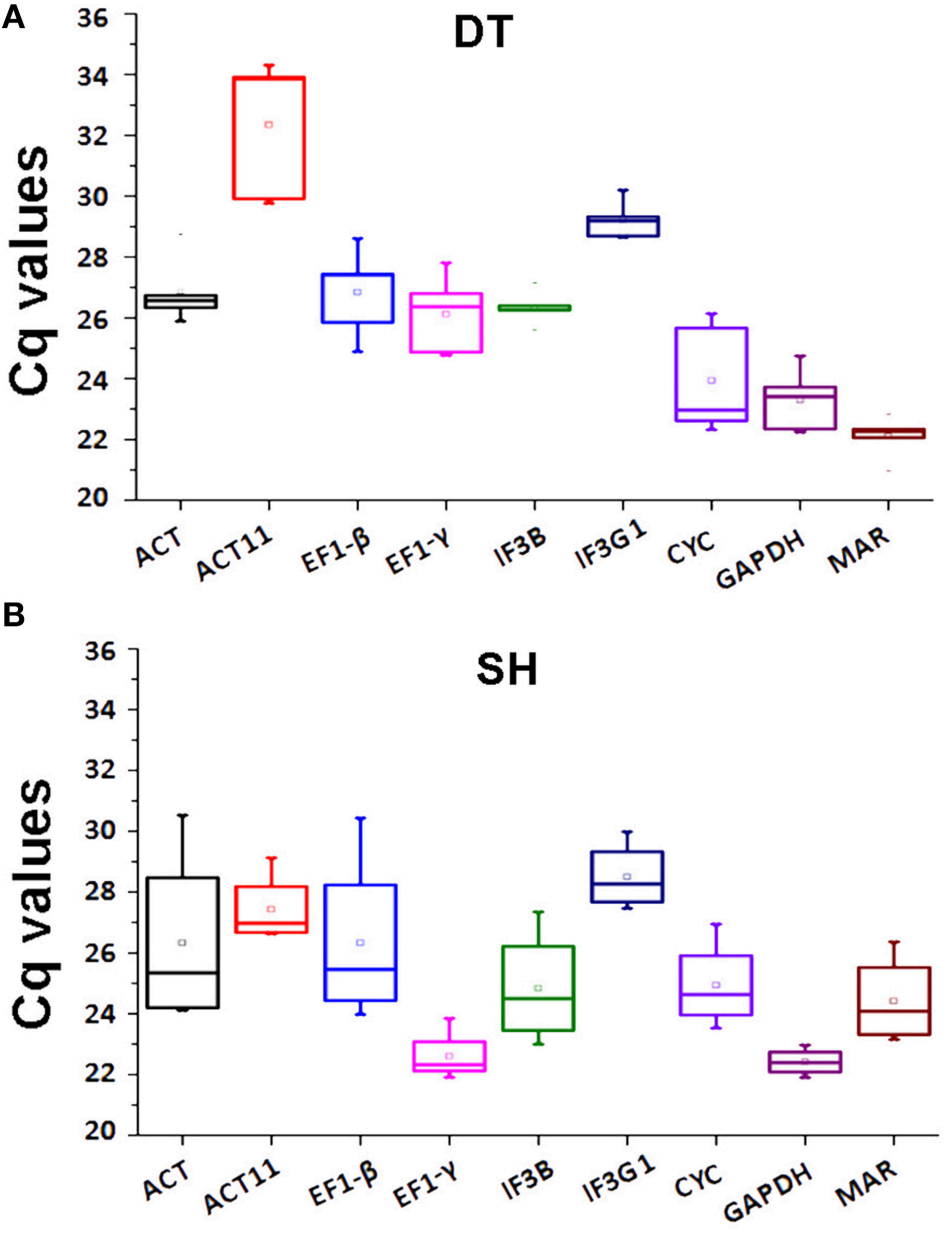
**Expression levels of eight candidate reference genes and ***MAR*** in DT or SH**. Box-plot graphs of Ct-values for each gene in DT **(A)** or SH **(B)** are shown. The plots highlight the mean (hollow square), median (center line), maximum and minimum (whiskers, the vertical lines), and 25 and 75% percentiles (boxes) of the data. The outliers are marked with dots. DT, different tissue sample group; SH, heat-treated seedling sample group.

The expression levels of eight tested candidate reference genes were determined using the threshold cycle (Ct)-values. The results showed that *ACT11* and *IF3G1* were the lowest expressed genes, with the highest mean Ct-values of 32.35 and 28.50 in DT and SH samples of *P. ginseng*, respectively. *GAPDH*, showing the lowest Ct-values of 23.30 in DT and 22.41 in SH, was the highest expressed gene in all tissues tested (Figure 2). Although each candidate reference gene showed expression variation in DT, *ACT11* displayed unacceptably high expression variation as shown in box-plots, where the boxes and whiskers of *ACT11* were the biggest among all tested candidate reference genes (Figure 2A). It suggests that *ACT11*, a most commonly used traditional housekeeping gene, is not suitable to be used as an internal control in RT-qPCR analysis of gene expression in given different tissues of *P. ginseng*. Similar results were also observed in rice (Jain et al., 2006). For SH samples, both *ACT* and *EF1-*β exhibited larger fluctuation than the other tested reference genes (Figure 2B), suggesting their instability in the samples tested.

### Expression stability analysis of reference genes in *P. ginseng*

**Figure 3:**
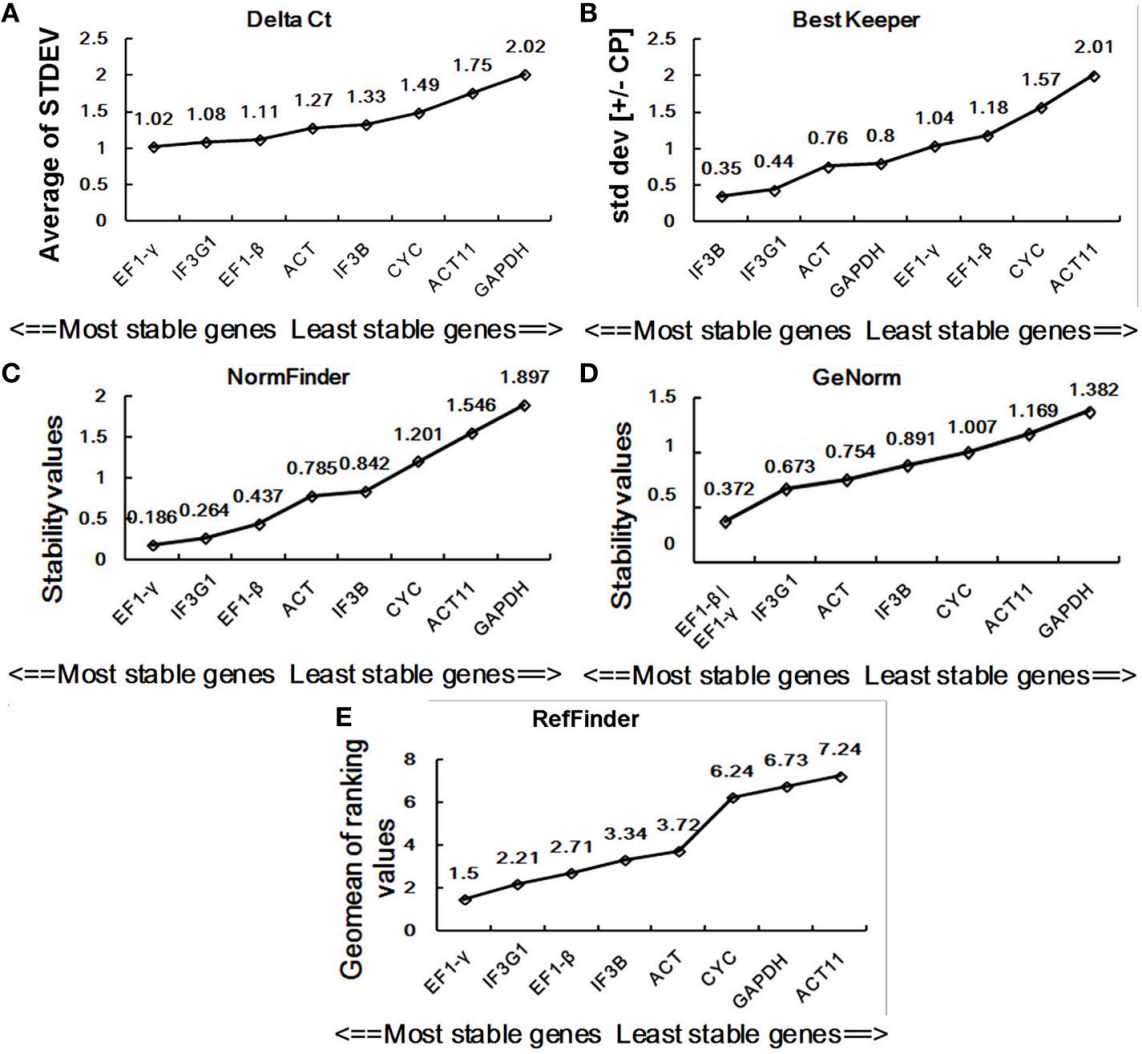
**Expression stability of eight candidate reference genes in DT**. Ranking was given by the comparative ΔCt method **(A),** BestKeeper **(B)**, NormFinder **(C)**, geNorm **(D)**, and RefFinder **(E)**. DT, different tissue sample group.

**Figure 4:**
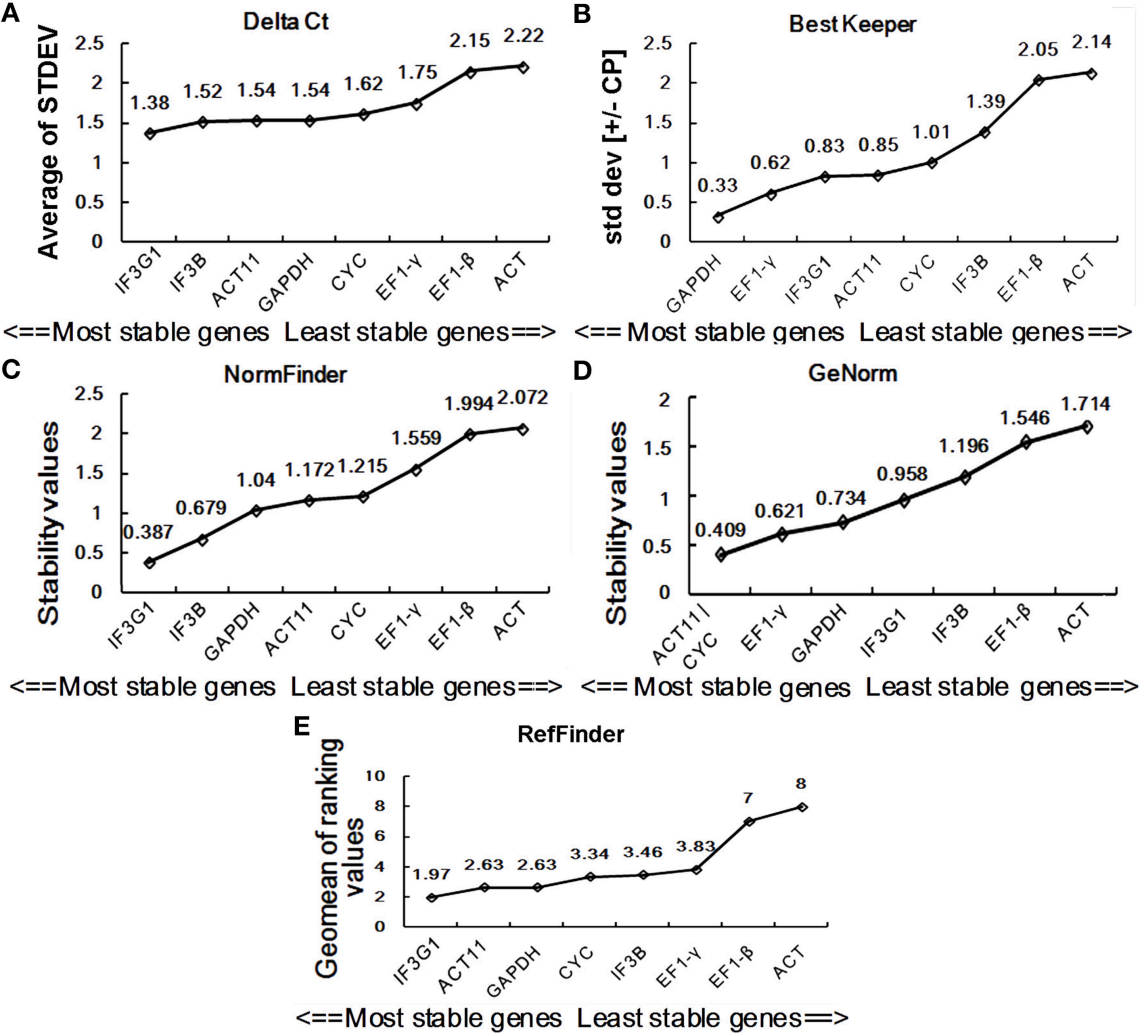
**Expression stability of eight candidate reference genes in SH**. Ranking was determined by the comparative ΔCt method **(A)**, BestKeeper **(B)**, NormFinder **(C)**, geNorm **(D)**, and RefFinder **(E)**. SH, heat-treated seedling sample group.

In this study, we used four major computational algorithms (the comparative ΔCt method, BestKeeper, Normfinder, and geNorm) to screen suitable reference genes. The methods can evaluate the expression stability of reference genes from different aspects. The comparative ΔCt method is a simple method by comparing relative expression of “pairs of genes” within each sample to confidently select useful housekeeping genes (Silver et al., 2006). According to this method, *EF1-*γ is the most stable reference gene showing the lowest standard deviation (STDEV) for the DT samples of *P. ginseng*, while *GAPDH* is the least stable one with the highest STDEV value (Figure 3A). For the SH samples, *IF3G1* showed the highest expression stability, while *ACT* was the lowest (Figure 4A).

BestKeeper is an Excel-based tool that estimates gene expression stability using the standard deviation (SD) and the coefficient of variation (CV) of each gene in different samples. Reference genes with SD >1 are considered unstable and should be avoided (Pfaffl et al., 2004). By BestKeeper, *IF3B, IF3G1, ACT*, and *GAPDH* presented stable expression with SD-values of 0.35, 0.44, 0.76, and 0.8, respectively, for DT samples (Figure 3B). For SH samples, *GAPDH, EF1-*γ, *IF3G1*, and *ACT11* are four stable reference genes with *SD*-values < 1 (Figure 4B). Since the best reference genes are those with the lowest CV and SD, *IF3B*, and *GAPDH* are the best reference genes for DT and SH by BestKeeper, respectively.

Normfinder can estimate not only the overall variation of the candidate reference genes but also the variation between subgroups of the total sample set (Andersen et al., 2004). A lower stability value indicates higher expression stability. For DT samples, Normfinder analysis showed a completely same pattern of stability with the comparative ΔCt method, recommending *EF1-*γ as the most reliable reference gene for RT-qPCR data normalization and *GAPDH* as the least one (Figure 3C). For SH samples, NormFinder also ranked *IF3G1* to be the best reference gene as the comparative ΔCt method. *ACT* showed unstable expression with a stability value of 2.072 (Figure 4C).

GeNorm has been widely used to determine the most stable reference genes from a set of tested genes by calculating a gene expression stability measure (M), the mean pairwise variation for a gene compared with all other tested genes (Vandesompele et al., 2002). Lower M-value is indicative of greater stability in expression. Ranking of the tested genes were given via a stepwise exclusion process. GeNorm analysis gives two most stable reference genes, since the analysis relies on the principle that expression ratio of two ideal reference genes must always remain constant across all samples. GeNorm analysis of candidate reference gene expression in DT samples showed that *EF1-*γ and *EF1-*β had the lowest M -value of 0.372, suggesting them to be the two most stable genes (Figure 3D). In SH samples, *ACT11*, and *CYC* had an M-value of 0.409, displaying the most stable expression (Figure 4D).

**Figure 5:**
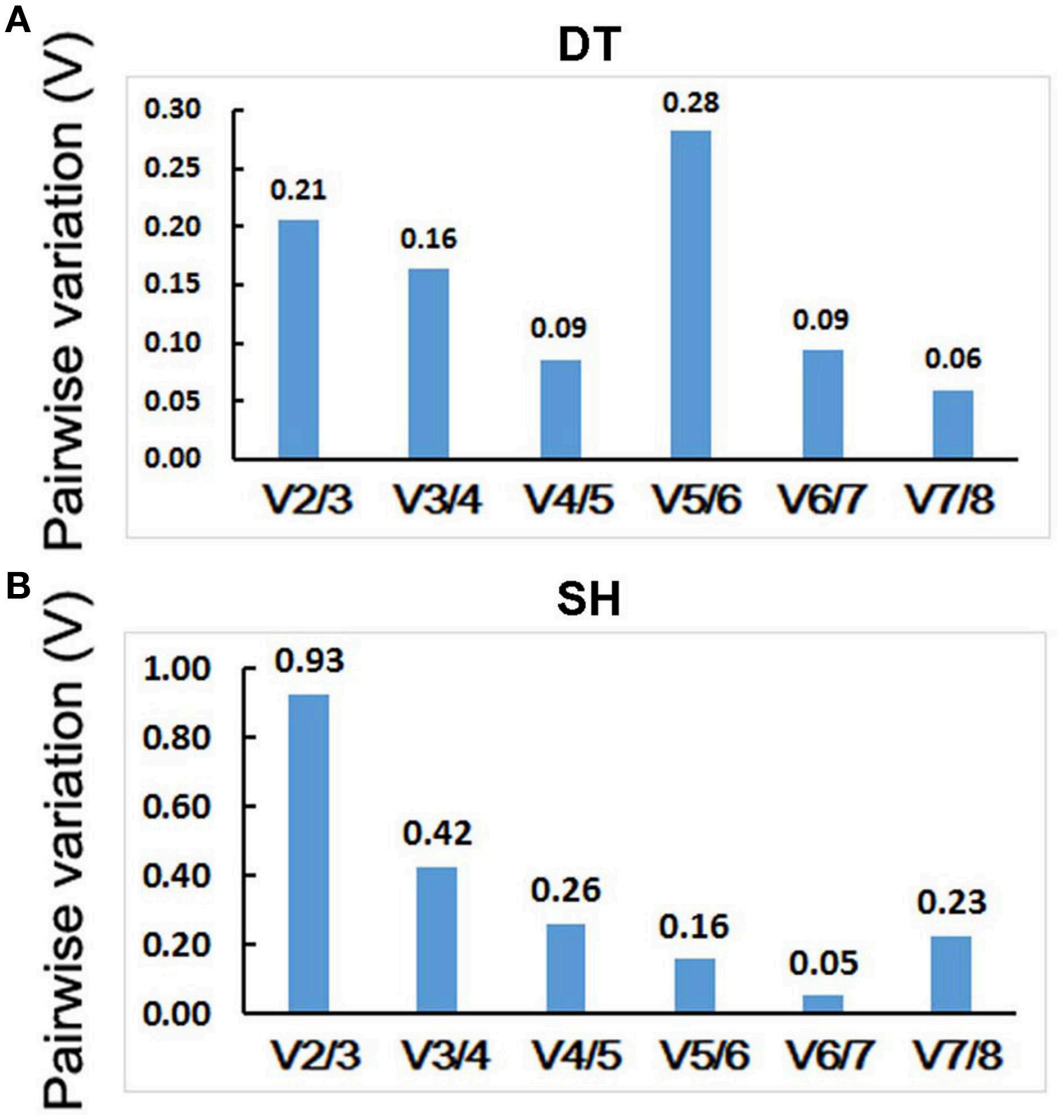
**Determination of the optimal number of internal control genes in DT (A) and SH (B)**. Pairwise variation (V) values were calculated by geNorm. No additional genes are required for the normalization when values below 0.15. DT, different tissue sample group; SH, heat-treated seedling sample group.

The geNorm program can also determine optimal number of reference genes for RT-qPCR normalization. It calculates pairwise variation of two sequential normalization factors NF_*n*_ and NF_*n*+1_ with a cut-off value of 0.15, below which the inclusion of an additional reference gene is not necessary. For *P. ginseng* tissues tested, normalization requires the use of four reference genes since only the V4/5-value (0.09) is inferior to the 0.15 cut-off level (Figure 5A). For heat treated seedlings, six reference genes were needed for accurate RT-qPCR data normalization since the V6/7-value (0.05) is < 0.15 (Figure 5B).

### Comprehensive ranking of the reference genes by RefFinder

Stability analysis of candidate reference gene expression using the comparative ΔCt method, BestKeeper, Normfinder, and geNorm showed different stability ranking list of genes (Figures 3, 4). For DT samples, GeNorm analysis showed *EF1-*γ and *EF1-*β to be two most stable genes; the comparative ΔCt method and NormFinder analysis suggested *EF1-*γ being the most ideal reference gene; while BestKeeper analysis ranked *IF3B* at the first position. For SH samples, GeNorm analysis showed *ACT11* and *CYC* to be top two ideal reference genes; the comparative ΔCt method and NormFinder analysis suggested *IF3G1* being the most stable gene; while BestKeeper analysis ranked *GAPDH* at the first position. Thus, a comprehensive evaluation of candidate reference genes were carried out using the web-based comprehensive tool RefFinder, which integrates geNorm, Normfinder, BestKeeper, and the comparative ΔCt method. As shown in Figure 3E, RefFinder analysis suggest that the ranking of expression stability is *EF1-*γ > *IF3G1* > *EF1-*β > *IF3B* > *ACT* > *CYC* > *GAPDH* > *ACT11* in the set of different *P. ginseng* tissues and *IF3G1* > *ACT11* > *GAPDH* > *CYC* > *IF3B* > *EF1-*γ > *EF1-*β > *ACT* in the heat-treated *P. ginseng* seedling set (Figure 4E).

### Expression analysis of *MAR* for reference gene validation

**Figure 6:**
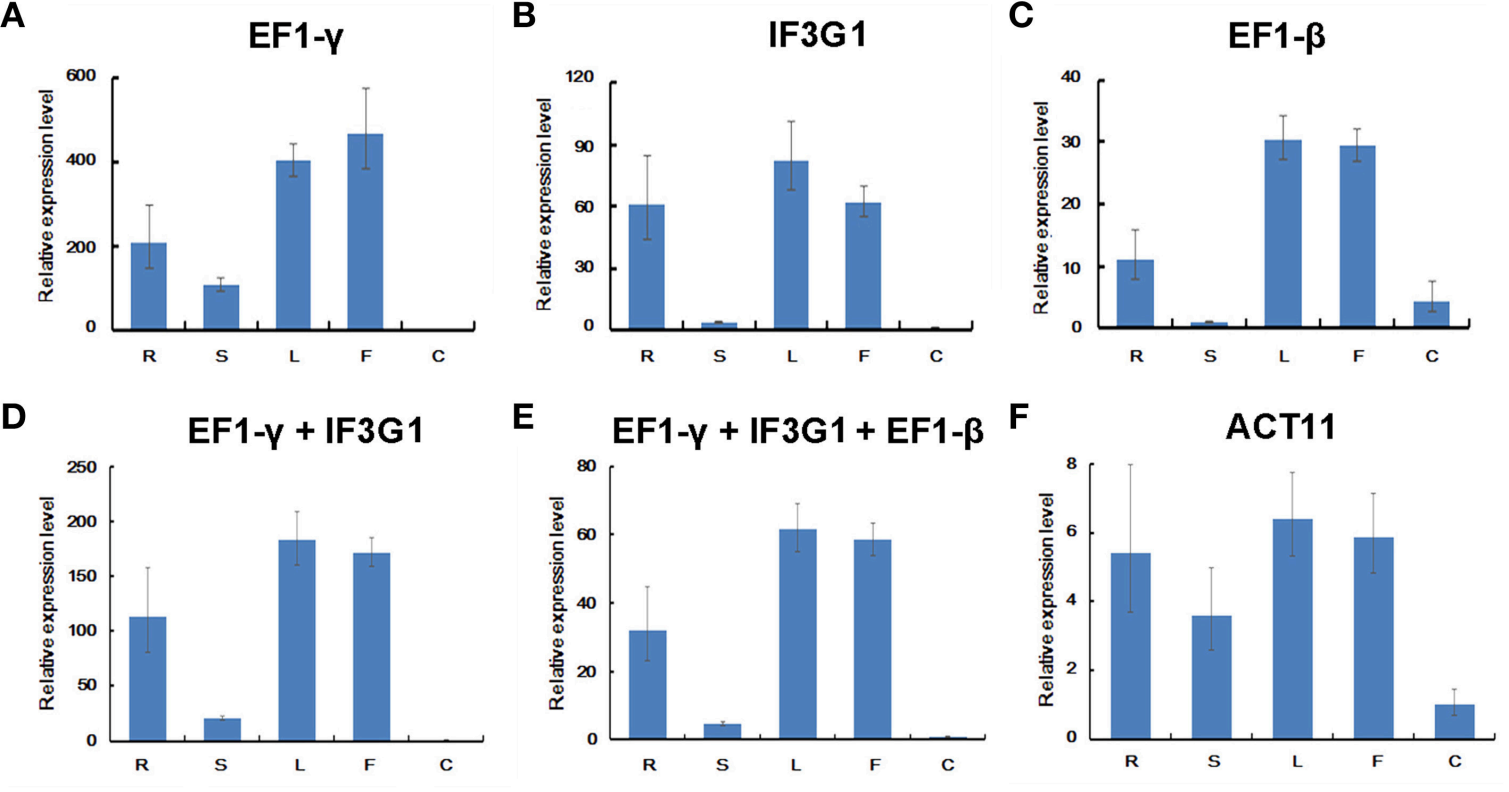
**Relative expression levels of ***MAR*** in ***P. ginseng*** tissues**. Different normalization factors were used: one of the three top ranked genes defined by RefFinder **(A–C)**, the combination of the two **(D)** or three **(E)** top ranked genes, and the worst stable gene **(F)**. All values are means ± SD (*n* = 3). R, root; S, stem; F, flower; L, leaf; C, embryogenic calli.

Previously, we analyzed the expression of a mlncRNA gene *MAR* using *GAPDH* for data normalization (Wang et al., 2015). However, *GAPDH* appears to be not most stably expressed in different tissues of *P. ginseng* in this study (Figure 3). Therefore, we used the three most stable genes, *EF1-*γ, *IF3G1*, and *EF1-*β shown by RefFinder analysis, as internal controls to re-analyze the expression pattern of *MAR* in *P. ginseng*. Although the overall expression profiles of *MAR* were similar, slight differences were observed when normalized using the three reference genes alone. For example, *MAR* transcripts accumulated at a higher level in flowers than leaves when the expression data was normalized using *EF1-*γ (Figure 6A). A lower or similar level of *MAR* was shown in flowers compared with leaves when *IF3G1* and *EF1-*β were used as the internal control, respectively, (Figures 6B,C). Using the combination of two (*EF1-*γ and *IF3G1*) or three (*EF1-*γ, *IF3G1*, and *EF1-*β) reference genes to normalize RT-qPCR data showed the same expression patterns of *MAR* (Figures 6D,E). Using the least stable gene *ACT11* as internal control, the expression of *MAR* pattern was not influenced (Figure 6F). But, the accumulation of *MAR* transcripts was under-estimated in roots when the data was normalized using *GAPDH* as internal control (Wang et al., 2015). It suggests that gene expression levels were obviously affected when unstable reference genes were used for normalization. Considering the cost and operation process, we recommend using the combination of *EF1-*γ and *IF3G1* to normalize RT-qPCR data of gene expression in different *P. ginseng* tissues.

**Figure 7:**
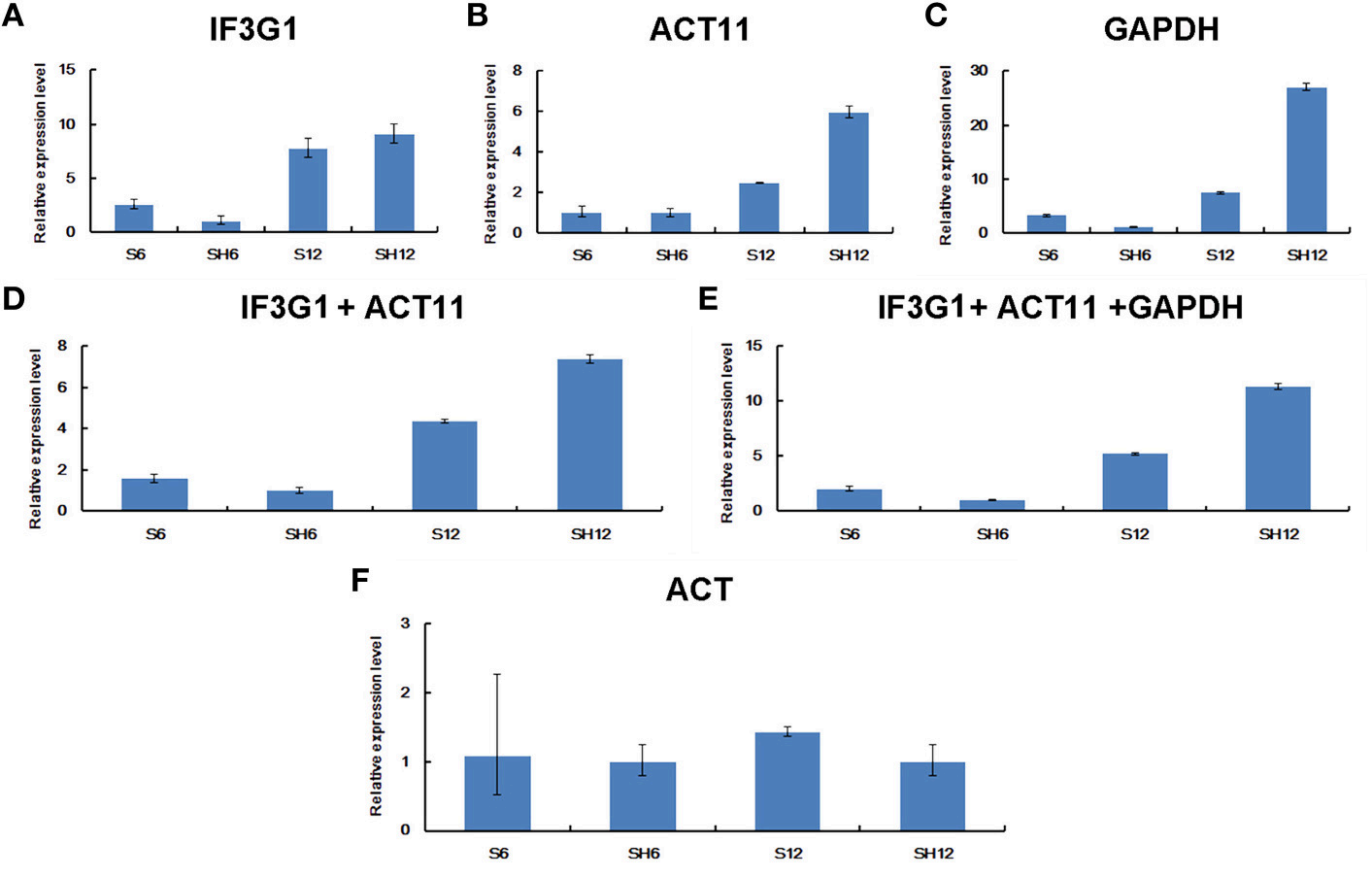
**Relative expression levels of ***MAR*** in ***P. ginseng*** seedlings under heat stress**. Different normalization factors were used: one of the three top ranked genes defined by RefFinder **(A–C)**, the combination of the two **(D)** or three **(E)** top ranked genes, and the worst ranked gene **(F)**. All values are means ± SD (*n* = 3). S6, seedlings grown at 25°C for 6 h; S12, seedlings grown at 25°C for 12 h; SH6, seedlings grown at 37°C for 6 h; SH12, seedlings grown at 37°C for 12 h.

Furthermore, we examined the expression of *MAR* in *P. ginseng* seedlings treated with heat stress. RT-qPCR data was normalized using the three most stable genes, including *IF3G1, ACT11*, and *GAPDH*, which were identified through RefFinder analysis. No matter normalized by *IF3G1, ACT11*, and *GAPDH* alone or a combination, nearly same expression profiles were obtained (Figures 7A–E). Compared with the level of *MAR* in seedlings without treatment, *MAR* expression was slightly down-regulated at the time-point of 6-h heat treatment, and then elevated at the time-point of 12-h treatment. On the contrary, down-regulation of *MAR* was shown when the data was normalized with *ACT*, the most unstable reference gene (Figure 7F). Taken together, we recommend using the combination of *IF3G1* and *ACT11* to normalize RT-qPCR data of gene expression in *P. ginseng* seedlings under heat treatment.

## Discussion

It is a crucial pre-requisite to validate appropriate reference genes for a successful gene expression measurement using RT-qPCR. An increasing number of studies on reference gene selection have been carried out in various plant species (Jain et al., 2006; Hong et al., 2008; Huang et al., 2014; Galli et al., 2015; Niu et al., 2015). As for *P. ginseng*, actin was frequently used as the internal control in RT-PCR (Lee et al., 2004; Han et al., 2006, 2010, 2011, 2012; Kim et al., 2011) or RT-qPCR (Kim et al., 2014) without any form of expression stability examination. Recently, Liu et al. (2014) carried out a systematic reference gene screening in different organs or at different developmental stages of *P. ginseng*. However, emerging evidence shows that the expression stability of reference genes may be altered across cultivars or under widely varying experimental conditions. For example, the traditionally used internal control genes *GAPDH* and *18S rRNA* were found to be the most unstable reference genes in given samples of strawberry cultivars or certain cultivars under various stress treatments (Galli et al., 2015). Thus, it is necessary to investigate the expression stability of candidate reference genes under specific experimental conditions prior to using them for RT-qPCR data normalization.

Selection of candidate reference genes for evaluation is the first step. It should cover a range of biological functions. We searched the ginseng transcriptome database using more than eight Arabidopsis commonly used reference genes by Blastx in order to select more candidates in the beginning. Unforturnately, most of them are short and is not suitable for primer design. In addition, there might be a big family for some candidate reference genes and they may share high sequence similarity. Therefore, genome-wide identification of all the members in each candidate reference gene family will be important for specific primer design of each gene. It is believed that the decoding of ginseng genome and the increasing transcriptome sequencing data will facilitate it.

Amplification efficiency is an important factor that should be considered when using a relative quantification approach for gene expression study. So far, two basic methods have been used for evaluation of amplification efficiencies. One uses a serial dilution of the cDNA samples and the Ct values were plotted against cDNA input. Then, the efficiency was calculated from the slope of the regression line based on the equation E = 10^(−1∕slope)^ (Pfaffl, 2001; Rasmussen, 2001). For the other one, amplification efficiency is computed for each gene from each individual reaction (Liu and Saint, 2002; Ruijter et al., 2009). It has been shown that amplification efficiency can vary largely depending on the approach used (Regier and Frey, 2010). We used the dilution series of pooled cDNA samples for efficiency calculation and not all of them were near 100% (Table 1). It is probably due to that the amplification efficiency is not constant for all dilutions. There may be chemicals in cDNA samples inhibiting the reaction. When a cDNA sample is diluted, these “poisons” are also be diluted and therefore, increases the amplification efficiencies in the diluted samples (Ramakers et al., 2003).

A good reference gene, which can be used as an internal control, is characterized by expression stability. The stability of expression is ideally not affected by tissue types, developmental stages, physiological situations, and experimental conditions. According to Liu et al. (2014), *CYP* (called *CYC* in our study) and *EF-1a* are the two most stable reference genes in different organs and developmental stages. However, we found that *CYC* was not the most stable in different tissues (Figure 3). This inconsistency is might be due to the quite different ginseng cultivars used. Similar results are obtained in strawberry and *18S* shows the most variation among different cultivars (Galli et al., 2015). It suggests that it is necessary to select suitable reference genes even among different cultivar. Interestingly, *EF1-*γ and *EF1-*β was found to be stable among different tissues by all the analysis methods except BestKeeper in our study (Figure 3). It is inferred that elongation factor genes might be stable among these two ginseng cultivars, even though further certification is needed.

Usually, only one reference gene was used for RT-qPCR data normalization in most reported gene expression studies (Suzuki et al., 2000). However, single reference gene is insufficient sometimes (Veazey and Golding, 2011). GeNorm recommended four and six reference genes for different tissues and heat treated seedling samples, respectively (Figure 5). Considering cost and operation, we analyzed the expression levels of *MAR* in different tissues of *P. ginseng* or seedlings treated with heat stress using the three most stable genes alone or a combination as internal controls. As a result, *EF1-*γ/*IF3G1* and *IF3G1*/*ACT11* were selected as the optimum pairs of reference genes for normalization of RT-qPCR data obtained from different tissues of *P. ginseng* and heat treated seedlings, respectively. The two gene pairs can serve as internal controls for not only mlncRNAs but also protein-coding genes gene expression studies.

In conclusion, this study evaluated the expression stability of eight candidate housekeeping genes in two set of tissues, including roots, stems, leaves, and flowers of 5-year-old *P. ginseng* plants and embryogenic calli of *P. ginseng* (DT) 1-month-old *P. ginseng* seedlings treated with or without high temperature for 6 and 12 h (SH). RT-qPCR data was analyzed using the comparative ΔCt method, geNorm, Normfinder, BestKeeper, and RefFinder. *EF1-*γ*/IF3G1* and *IF3G1/ACT11* were identified as the optimum pairs of internal control genes in different tissues of *P. ginseng* or seedlings under heat treatment, respectively. The results provide useful information for reliable RT-qPCR data normalization in *P. ginseng* gene expression studies.

## Author contributions

Conceived and designed the experiments: SL. Performed the experiments: MW. Wrote the paper: MW and SL.

### Conflict of interest statement

The authors declare that the research was conducted in the absence of any commercial or financial relationships that could be construed as a potential conflict of interest.
