# Supplementary material for: Validation of Suitable Reference Genes for Quantitative Gene Expression Analysis in Panax ginseng
Source: Front Plant Sci. 2016 Jan 12;6:1259. doi: 10.3389/fpls.2015.01259 (PMC4709418; doi:10.3389/fpls.2015.01259)
Supplement: Supplementary file 1 [file Image1.PDF]

Supplementary Information

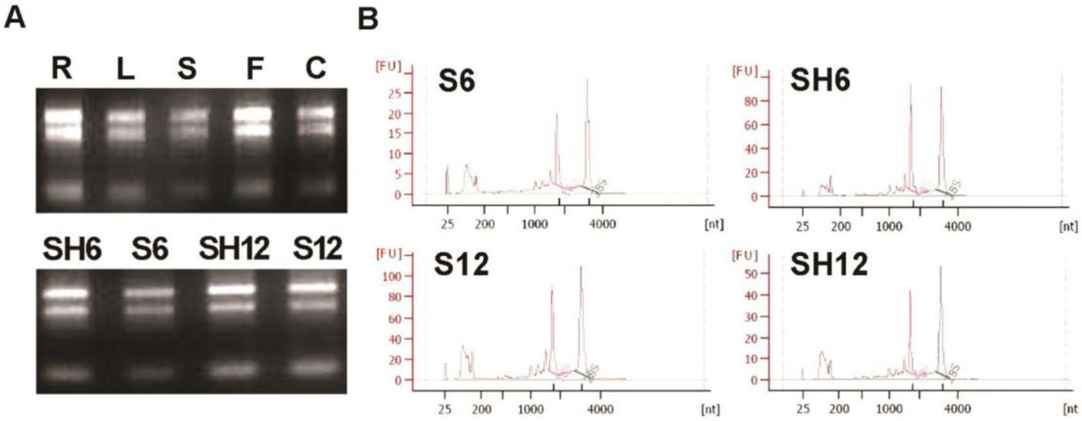

C

RNA quantity and quality measurement

Samples	Concentration (ng/μl)	RIN	$OD_{260/280}$	$OD_{260/230}$
R	1153	-	2.08	2.12
L	609	-	2.02	2.03
S	772	-	2.07	2.1
F	1045	-	2.09	2.1
C	834	-	2.06	2.1
S6	276	8.0	2.05	2.08
SH6	1512	8.1	2.07	1.8
S12	1008	8.1	2.10	2.01
SH12	984	8.0	2.06	1.8

\- Not examined.

Figure S1. The quality and quantity of total RNA samples used in this study. (A) Agarose gel (0.8 %) electrophoresis of total RNA from each sample. (B) Quality assessment of total RNA from heat stress seedlings using Agilent 2100 (Agilent Technologies, Palo Alto, CA). Results showed that they had a RNA integrity number (RIN) of >8.0, suggesting intact RNA samples. (C) Summary of RNA quantity and quality used in this study. The concentration of total RNA was examined by NanoDrop-2000C spectrophotometer (Thermo Scientific, Wilmington, DE).
