# Supplementary material for: Validation of Suitable Reference Genes for Quantitative Gene Expression Analysis in Panax ginseng
Source: Front Plant Sci. 2016 Jan 12;6:1259. doi: 10.3389/fpls.2015.01259 (PMC4709418; doi:10.3389/fpls.2015.01259)
Supplement: Supplementary file 2 [file Image2.PDF]

A

>EF1-β

ACCCCTAGCACTCTCTAGGTTTCCGTCTCCCTTTCTTCGTCGCAGCTCTTCTTTTCATCATCAAACTAGAAAATGACTGTTACATTCACCGATTTGCATACCGAATCTGGACTTAAATCTCTCAATGAGTTT
CTTTCGGGAAAAACCTGCATCTCTGGGGATAAGCTTACAAAGGATGATGTAAAGGTTTATGCAGCTGTTTTGGAGAAGCCCAGTGGTGATCTCTTCCCGAATGCAAGTCAGTGGTACGACAGTGTTTCTTC
CCAACTCGCCGCAAGTTTCCCTGGTACAGCTATTGGTGTAAGAATTGCCAGCCATTCTGCTCCAGCAGAAGCTACTCCAGCTAAGGAAGCTGAAAAGGAATCTCCTGCTGAAGATGATGATGACCTTGATC
TGTTTGGTGATGAGACCGAGGAGGAGAAGGCGGCGGCAGAACAGAGAGAGGCATCAAAAGCATCCTCCAAGAAGAAAGAGAGTGGGAAATCATCTGTTCTTTTGGATGTGAAGCCTTGGGACGATGAGACA
GACATGAAGAAATTGGAAGAGGCTGTTCGCAGTGTTGAAATGCCCGGGCTCTTTTGGGGAGCTTCAAAACTACTTCCTGTTGGTTATGGGATAAAGAAAATGACTATCATGCTGACCATTGTGGATGACCT
TGTGTCCGTTGATTCTCTCATAGAGGAGCATCTCACAGTTGAGCCTATAAATGAATATGTACAGAGCTGTGATATTGTTGCCTTCAACAAAATCTGAGAAGAACTTGGTAGAATTGTTCCAGACTTTGCCC
CATTCCAGGCAGTAATTTTGTTACGTTGTAGTCGTGATTGTTCTTGGTTTAAGTTATTTTTTGTGAGTTGTATGATTTGTCCTAGAAAGGACTCATGTTTTAAAATGTCTTGACTGGAAACATTTGATGTTG
AGGAGCTTAATTTCATGTTAATGACTATCGTGACCTTGTGATTGATT

B

>EF1-γ

GGAGCCAACGGCGGCTGTTTCACAGCAGATTTGATCGTAAAAGGAGGGAACAAGCTCTCACTGTGAAGCCATGGCTCTGATTTTGCATGCGGGGAACCCAAATAAAAATGCTTGGAAGGCACTCATAGCGGC
AGAATACAGTGGTGTGAAAGTTGAACTGGTCAAGGATTTTCAGATGGGTGTATCGAACAAAACGCCAGAGTTTCTCAAAATGAACCCTATGGGGAAGGTTCCAGTGCTTGAAACACCTGATGGTACTGTTT
TTGAGAGTAATGCCATAGCACGTTATGTTGCTCGTGTGAAGCCTGATAATTCCCTTTTTGGATCTTCGCTGATTGATTATGCCCACATTGAGCAATGGATTGATTTTGCATCGTTGGAGATTGATGCCAAT
ATTGCGCGATGGTTATATCCACGAATGGGTTTTGCTATTTACCTTCCTCCGGCGGAAGAAGCTGCAATCGCATTAAAGAGAGCACTAGGAGCTTTAAATACCCATCTTGTTTCCAACACTTACTTGGTTGG
GCATTCCGTCACTCTGGCTGATATTATTATGACATGCAACTTGGCTTTGGGATTTAATCGAATTTTGACCAAGAGCTTTACCACAGAATTTCCACACGTAGAGAGATATTTTTGGACCATGATTAATCAAC
CAAACTTCTCCAAGATATTGGGTGAGCTCAAACAAGCAGATTCTGTACCACCTGTTCAGTCAGTGAAGAAGCCCGCACAACCTAAGGAACCTGCAAAATCTAAGGTTCAGGATGAACCTAAGAAAGAGGC

C

>IF3G1

GGAAAAGGTGGTGCTGTTCTCATGGTATGCAGAACTTGTGGCAAGAAAGGTGATCACTGGACATCTAGATGCCCTTACAAGGATCTTGCCCCACAGGCTGATGTTGATAAACCCCCAACCTCGGATACAAC
AATGGCTTCTGGCACCACAAAGGGAGCTTATGTTCCCCCAAGCATGAGAGCAGGTGCAGAGAGACCTGTAGGGTCTGATATGAGACGCAGGAATGAGGAGAACTCAGTTCGGGTCACCAATTTATCAGAAG
ACACAAGAGAACCAGACTTGCTTGAGCTATTCCGCACATTTGGCCCTGTGAGTCGTGTCTACGTCGCCATTGATCAGAAGACAGGTGTCAGCAGGGGTTTTGGTTTCGTCAACTTTGTAAGCAAGGAAGAT
GCAGAGAGAGCCATCATGAAGTTGAATGGATATGGGTATGACAATTTAATCCTTCGTGTGGAGTGGGCCACACCAAGGGCGAACTAGAATATTTTCATCTCCAGCAGAATTTCATGTCAATGGTTTTGGTCA
GAGTTGCATCCAATATGCTGCTGATATGCTGTTTGTTTGTTGCTTCAGTATTTCATTGTCTTTTTTGTCCCCACTGTTTAGGTTTAAGAGTTAAGTATCCATATTTTGGACTAGATACTTATATTATCAATG
GAATTGTTATCCCTTCTCTTCTTTTAATAGAACCCAGCTACTGTCATCTTTTGCTTTAGGAATTTATGGACTTGACCAGGGTATCATAATTTTGCAGTTTGCAAGTACTGGTTCAAAGACTGTACTTGTACT
TTATGC

D

>IF3B

CACACACTCCCTCTAGCGAGCCAAGAGCTCTGTGAAGCTAGCAGCATCAAACCCCCCCTTTTTTTCTGTATTGAGACCAGCAAAATCTGGTCTCTTAATCTCTCTCCTTTCTGCAATCATGGCGGATACAAT
TTCTATGGAGGACATCCGAGCCACAGCTGGTAGCCTCGGAATTGACCTCTCCAGCGTCGATTGGGACTCAATTCACCTTCCTCCTGGTGAAGATTTTGGCATAAAAAGTGACGATGAGGATCTTTATGAAG
AGGATTCGATGGAATTTGAAGGCGGATTTGGTAACATAATTGTTGTGGATAACTTACCGGTTGTTCCGAAAGAGAAATTTGAAAAATTGGAAGGAGTGGTCCGTAAGATTTACAGCCAAATTGGTGTGATT
AAAGAGAATGGGCTATGGATGCCCGTTGATCCTTCCACTCAGAAAACCCTTGGTTACTGCTTCATTGAGTATAACACTCCTCAGGAAGCAGAGCTCGCAAAGGAGAAGACAAATGGATACAAGTTAGACAG
GGCACACGTATTTGCTGTGAACATGTTTGATGAAATAGAGAAGTTCATGAAAGTTCCAGATGAATGGGCCCCTCCAGAAACCAAGCCATATACATCAGGGGAAAATTTACAACACTGGCTTACTGATGAGA
AAGCTAGAGATCAGTTTGTTATTCGTGCTGGCTCGGTCACAGAGGTTTTGTGGAATGATGCCAGACAGTTGAAGGCTGATCCTGTTTACAAACGTGATTACTGGACTGAAAGTTTTGTGCAGTGGTCCCCT
TTAGGGACCTACTTAGCCACAGTTCATAGGCAGGGAGCTGCTGTTTGGGGTGGGGCTTCTACCTTTAATCGTCTTATGCGTTATGCTCATCTGCAGGTAAAATTGATTGATTTTTCTCCTGGCGAGAGATT
TTTGGTGACTTACAGCAGCCATGAGCCAAGCAATCCTCGTGATACTCATAGGGTTGTGCTAAATATATTTGACGTGAGAACTGGAAAAGTAATGAGAGATTTCAAAGGAAGCGCAGATGAGTTTGCAGTTG
GGGGAACTGGAGGTTTTGCTGGGGTCTCCTGGCCTGTTTTCAGATGGGGTGGTGGAAAAGATGATAAGTACTTCGCTAGAATTGGCAAAAATGTCATTTCTGTTTATGAAACTGAGACATTTGCTCTTATT
GAAAC

Figure S2. Nucleotide sequences of *EF1-β* (A), *EF1-γ* (B), *IF3G1* (C) and *IF3B* (D).

The coding regions are indicated in bold, the start or stop codons are signed in box, and the primer pairs are marked in red (forward) or in blue (reverse).
