# Supplementary material for: Validation of Suitable Reference Genes for Quantitative Gene Expression Analysis in Panax ginseng
Source: Front Plant Sci. 2016 Jan 12;6:1259. doi: 10.3389/fpls.2015.01259 (PMC4709418; doi:10.3389/fpls.2015.01259)
Supplement: Supplementary file 3 [file Image3.PDF]

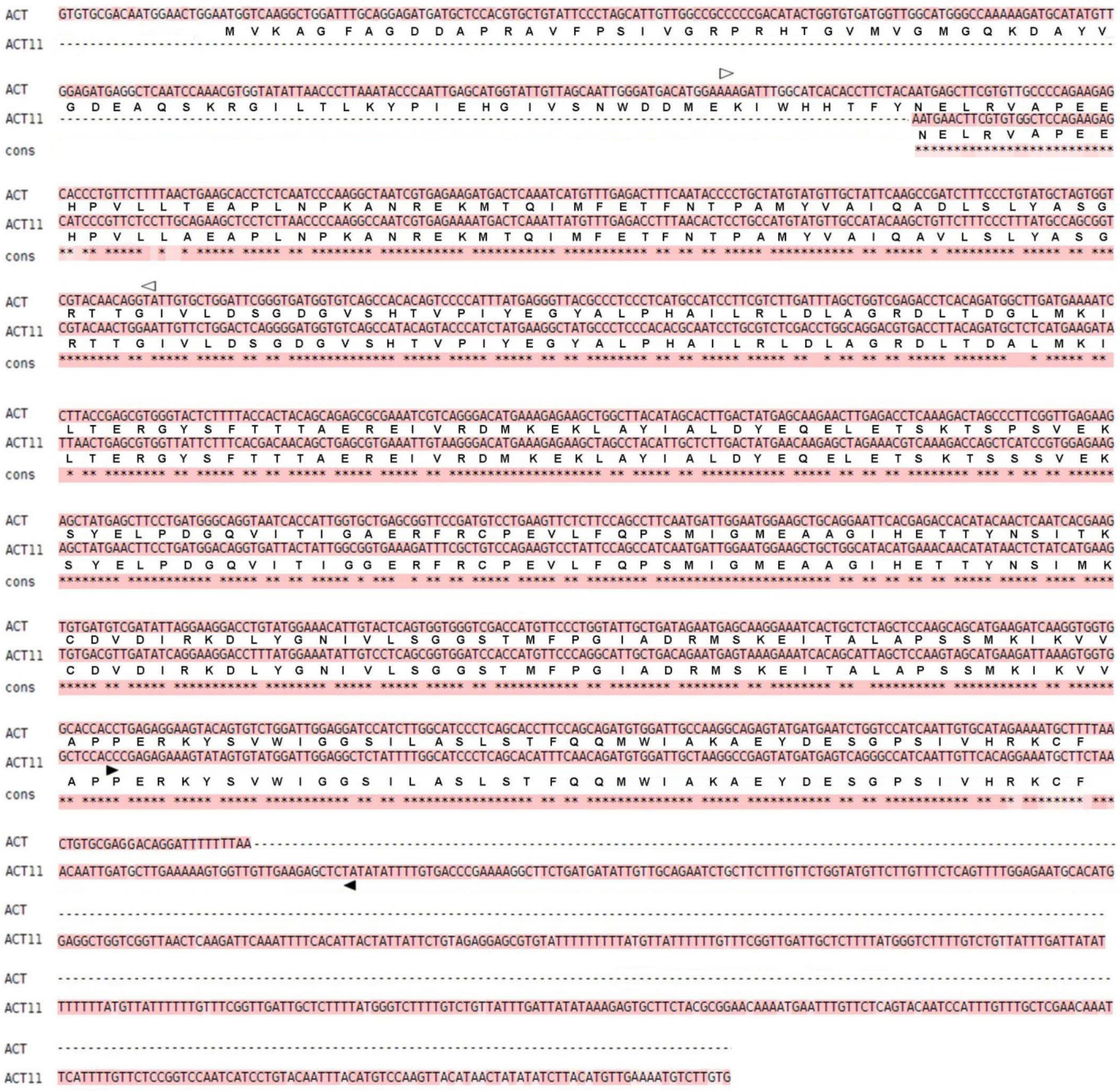

Figure S3. Sequence alignment of *ACT* and *ACT11* by T-Coffee. The amino acids are indicated below the nucleotide sequence. Identical nucleotides are shown in star. The primer pairs of *ACT11* and *ACT* were indicated with black or white triangles in (A), respectively.
